# Computational modeling reveals molecular details of epidermal growth factor binding

**DOI:** 10.1186/1471-2121-6-41

**Published:** 2005-11-30

**Authors:** Kapil Mayawala, Dionisios G Vlachos, Jeremy S Edwards

**Affiliations:** 1 Department of Chemical Engineering, University of Delaware, Newark, DE, USA; 2 Molecular Genetics and Microbiology, Cancer Research and Treatment Center, University of New Mexico Health Sciences Center, and Chemical and Nuclear Engineering, University of New Mexico, Albuquerque, NM, USA

## Abstract

**Background:**

The ErbB family of receptors are dysregulated in a number of cancers, and the signaling pathway of this receptor family is a critical target for several anti-cancer drugs. Therefore a detailed understanding of the mechanisms of receptor activation is critical. However, despite a plethora of biochemical studies and recent single particle tracking experiments, the early molecular mechanisms involving epidermal growth factor (EGF) binding and EGF receptor (EGFR) dimerization are not as well understood. Herein, we describe a spatially distributed Monte Carlo based simulation framework to enable the simulation of *in vivo *receptor diffusion and dimerization.

**Results:**

Our simulation results are in agreement with the data from single particle tracking and biochemical experiments on EGFR. Furthermore, the simulations reveal that the sequence of receptor-receptor and ligand-receptor reaction events depends on the ligand concentration, receptor density and receptor mobility.

**Conclusion:**

Our computer simulations reveal the mechanism of EGF binding on EGFR. Overall, we show that spatial simulation of receptor dynamics can be used to gain a mechanistic understanding of receptor activation which may in turn enable improved cancer treatments in the future.

## Background

Amplification of genes for the ErbB family of receptors is associated with poor outcome in women's cancers, including breast, ovarian and endometrial cancer. Under non-pathological conditions, epidermal growth factor (EGF) receptor (EGFR) or ErbB1 is activated by ligand-induced receptor dimerization, resulting in autophosphorylation and phosphorylation of various cellular substrates [1]. However, while it is clear that overexpression is a factor leading to ligand-independent signaling via these receptors, the mechanism by which functional dimerization and activation occurs is unknown. Since EGF binding represents the initial step for activating EGFR, considerable work has been devoted to elucidating the mechanisms of ligand binding and dimerization [1-7]. However, molecular details of ligand-induced receptor dimerization are not as well understood.

Apart from *in vitro *biochemical experiments to study mechanisms of EGFR activation [1], recent developments in microscopy have made it possible to visualize protein dynamics in living cells [8]. The current imaging methods either have a high spatial resolution, such as electron microscopy experiments using immunogold labeling [9] and covalent linking to chemical conjugates like ferritin [10], or high temporal resolution, such as fluorescence confocal microscopy [11], single particle tracking [5] and more recently quantum dots based ligands [12]. However, with the currently available imaging technologies, combined high temporal and spatial resolution (of multiple receptors) has not been achieved.

Computational efforts devoted to understanding the extracellular mechanisms leading to EGFR activation are mostly equilibrium studies [7,13-15] or continuum reaction-diffusion models; see references in [3,16]. Continuum partial differential equation based models have also been used to represent signaling processes in the plasma membrane assuming a continuum distribution of receptors [17].

While such studies have provided useful insights, they are not ideally suited for describing cell surface heterogeneities, such as microdomains and anomalous diffusion of surface receptors [18], which are important to capture the spatiotemporal receptor dynamics, and lack spatial correlations, known to arise from bimolecular reaction events [19], such as dimerization. Monte Carlo (MC) techniques have proven powerful for systems biology modeling [20-22]. In the past, spatial MC approaches have provided mechanistic understanding in other biological systems; see for example [20,23-33].

In this study, we have used a spatial MC framework which not only enables a realistic representation of the plasma membrane, but also facilitates integration of different types of biological data produced from biochemical and microscopy studies to gain insight into the mechanistic details of the underlying biological process. We have developed a general kinetic, lattice MC modeling framework to model the ligand (EGF) binding and dimerization of the EGFR. We compare our simulation results with single particle tracking experiments and analyzed the dominant mechanism of ligand binding and dimerization.

## Results

### Comparison of stochastic and deterministic models

**Figure 1:**
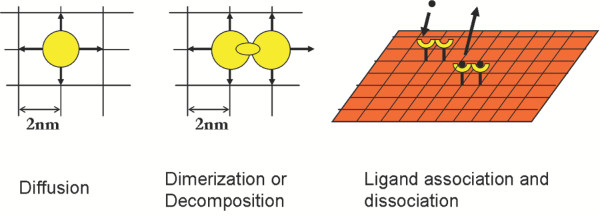
Schematic of simulated microscopic events. Each receptor can diffuse to an empty neighboring site, react with a neighboring receptor to form a dimer, and bind ligand. All events are reversible.

**Figure 2:**
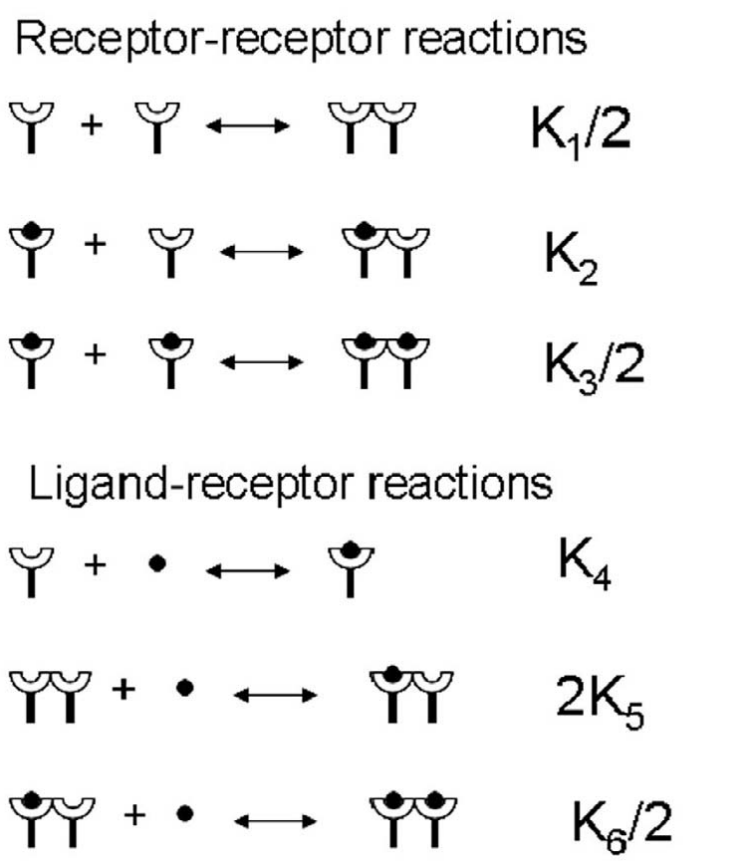
Reactions events considered in our model as given in [14].

**Figure 3:**
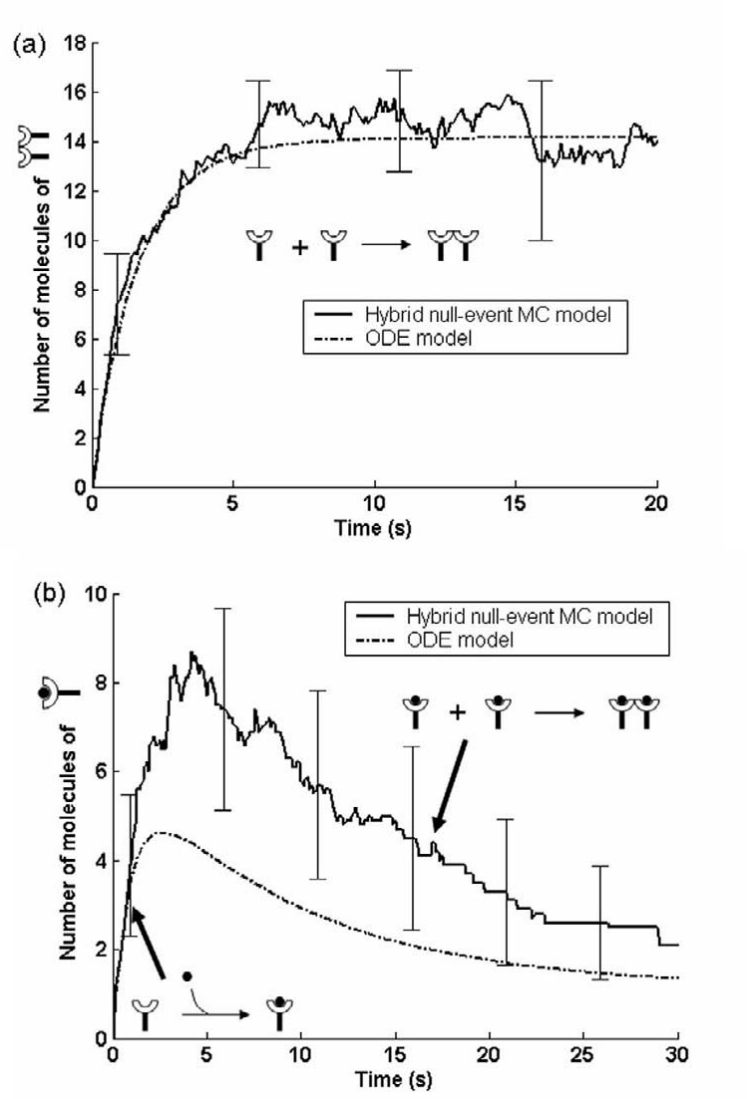
Comparison of hybrid null-event MC and ODE models in terms of (a) dimerized EGFR in the absence of ligand at a high receptor density and diffusivity (11,000 per μm^2^, D = 2 × 10^-14 ^m^2^sec^-1^) and assuming no initial dimers, and (b) EGF bound EGFR in the presence of ligand (160 nM) at low receptor density (125 per per μm^2^) and D = 2 × 10^-15 ^m^2^sec^-1^. The reactions on the figure indicate the dominant processes responsible for the concentration trajectories. Error bars indicate 2 standard deviations obtained from 10 independent MC simulations.

Microscopic events modeled in this work are shown in Figs. 1 and 2. In order to test the MC algorithm and explore possible differences between stochastic and deterministic models, we have performed a number of simulations for various parameters. Results from the hybrid null-event MC algorithm were compared with an ordinary differential equations (ODEs) model with a set of parameters for which the process is reaction limited, i.e., diffusion is fast compared to reaction, in order to test the validity of the MC algorithm. Specifically, we simulated a dimerization reaction in the absence of ligand considering a high receptor number density of 11,000 per μm^2 ^and assuming no dimers initially. Fig. 3(a) compares the concentration trajectories of dimerized EGFR. This comparison confirms that the hybrid null-event MC algorithm captures the time scales of the system resulting in the correct transient concentration profile. Additional validation carried out under diffusion control has again demonstrated the accuracy of our MC method (Mayawala *et al*., in preparation).

Next in Fig. 3(b) we compared the MC and ODE concentration profiles of EGF bound EGFR monomer in the presence of ligand (160 nM), with a receptor number density of 125 receptors per μm^2 ^and a low diffusivity of 2 × 10^-15 ^m^2^s^-1^. The low values of receptor density and diffusivity result in a diffusion controlled case. Corresponding to these parameters, the receptor dimerization rate in the spatial MC model was slower compared to that of the ODE model. The diffusion limited dimerization of EGF bound EGFR monomer leads to a higher concentration of unbound receptor in the spatial MC model than in the ODE model. Thus, spatiotemporal MC simulations are required to capture the transient concentration profiles of the signaling species under diffusion limited conditions. Overall, low receptor densities and low diffusivities may render the system diffusion limited. Under such conditions, well-mixed simulations do not provide accurate dynamics. Use of spatial MC bypasses the question whether the system is diffusion or reaction limited. In a forthcoming communication, we will address quantitatively the conditions for which spatial MC simulations are needed.

Partial differential equations (PDEs) have traditionally been used to model diffusion-reaction processes when spatial effects become important. However, accurate representation of receptor-receptor reactions typically requires MC simulation due to the spatial inhomogeneous distribution of receptors stemming from spatial correlations [19,28]. Aside from spatial correlations, realistic representation of the plasma membrane microdomains and anomalous diffusion make MC simulation indispensable [18]. Due to these limitations, PDE models have not been employed here.

### Comparison of hybrid null-event MC simulations with single particle tracking experiments

**Figure 4:**
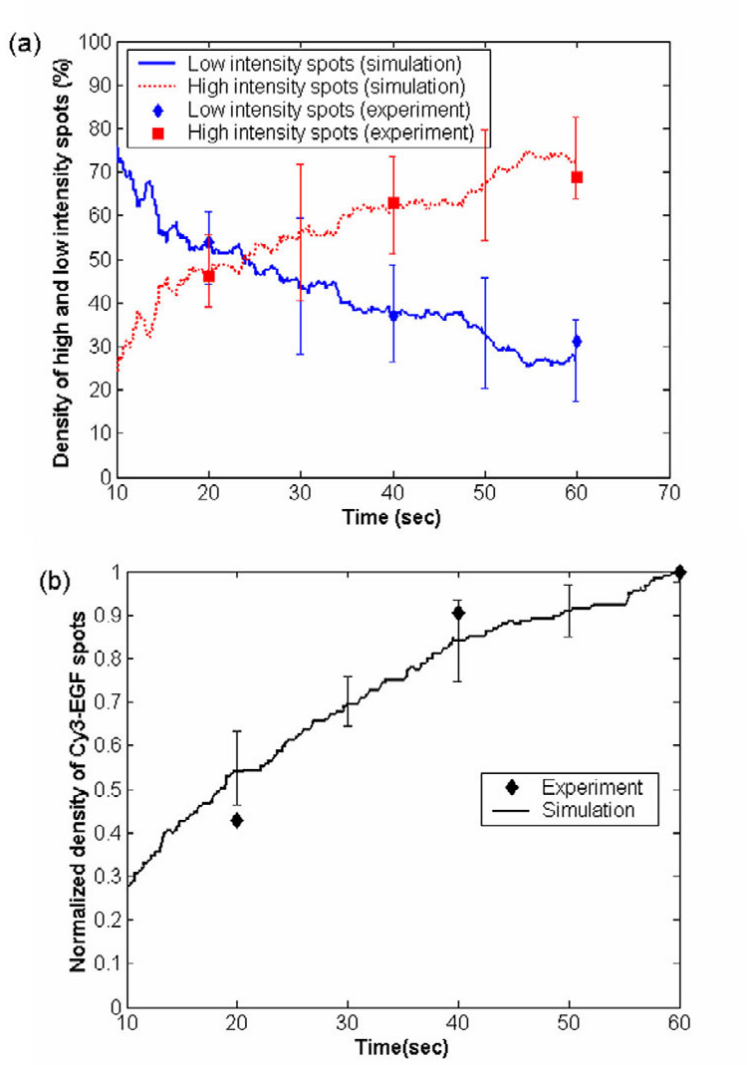
(a) Evolution of intensity of dimerized receptors with two ligands (high intensity spots) and of monomer plus dimerized receptors with a single ligand bound (low intensity spots) along with the data of single particle tracking experiments by Sako *et al*. over time intervals of 20 sec. The simulations were performed for a receptor number density of 5500 per μm^2^, a diffusivity of D = 2 × 10^-14 ^m^2^sec^-1^, and 18% dimers initially. The simulation intensity has been normalized with the experimental data. (b) Comparison of predicted density of Cy3-EGF spots with experimental data of Sako *et al*. The densities are normalized with the value at 60 sec. Good agreement of simulations with experimental data is found. In both panels, error bars indicate 2 standard deviations obtained from 10 independent MC simulations.

The dynamics of the ligand binding events were compared with the single particle tracking experiment of Sako *et al*. [5] at an EGF concentration of 0.16 nM in the 0–60 sec time interval. To compare simulation results with experimental data, EGF was assumed to be associated with Cy3 dye. A dimerized receptor with two EGF molecules was taken to fluoresce twice as intensely as a receptor (single or dimerized) bound to one EGF molecule. The predicted initial increase of low intensity spots (monomers plus dimers having one EGF bound) followed by a slower increase in high intensity spots (dimers with 2 molecules of EGF) is qualitatively consistent with the experimental data (see Fig. 4(a)). The initial increase in the low intensity signal was due to the rapid binding of EGF on predimerized EGFR. Furthermore, the increase in the total density of Cy3-EGF spots (total bound EGF on all receptors), shown in Fig. 4(b), is also consistent with experimental data.

**Figure 5:**
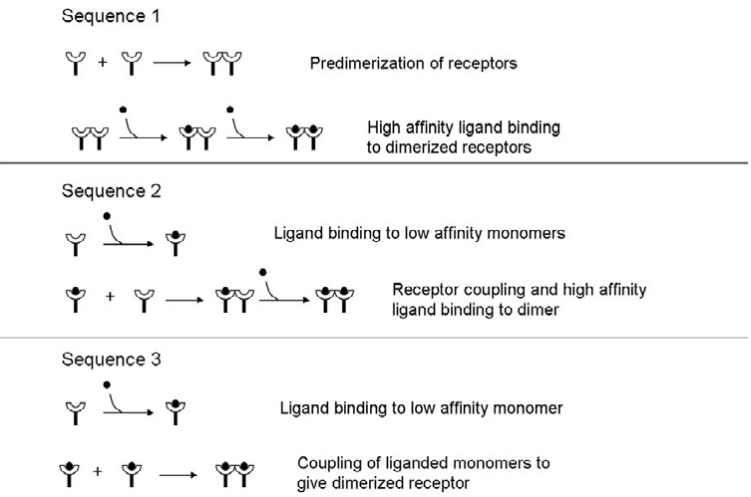
Sequence of reactions resulting in dimerized receptors with both receptors bound to ligand for simulations of Fig. 4. All reactions are reversible.

The possible sequences of events leading to the formation of EGF bound dimerized EGFR at 60 sec are shown in Fig. 5. Sako *et al*. [5] suggested sequence 1 as being dominant. However, the experimental study alone cannot unambiguously determine the sequence due to its limited spatial resolution and the fact that only ligand bound receptors can be tracked. Our simulations showed that 95–100% of the receptors follow sequence 1, 0–4.9% sequence 2, and the remaining receptors follow sequence 3. Our results are consistent with the hypothesis of Sako *et al*. [5]. This comparison serves as a model validation step. Small adjustments (20–30%) in the equilibrium and kinetic parameters tabulated in Table 1, which are well within the margins of error, lead to nearly proportional changes in intensity, i.e., no dramatic differences in the simulation profiles are seen (see appendix for details).

**Table 1:** Kinetic (reaction events given in Fig. 2) and transport (monomer and dimer diffusion) parameters used in hybrid null-event MC model (factors of 1/2 and 1/4 discussed in the Methods section have to be considered).

**Equilibrium constants**		
K_1_	13.3 (molecule/site)^-1^	In the range to be consistent with ~18% of the monomer EGFR as dimers in the absence of EGF [9, 53–56]
K_2_	4.0 × 10^3 ^(molecule/site)^-1^	Calculated based on equilibrium relations given in [14]
K_3_	1.2 × 10^6 ^(molecule/site)^-1^	Calculated based on equilibrium relations given in [14]
K_4_	4 × 10^8 ^M^-1^	In the range suggested by [1, 4, 58–60]
K_5_, K_6_	1.2 × 10^11 ^M^-1^	In the range suggested by [9, 53–56]

**Kinetic parameters**		

k_1b_, k_2b_	0.17 sec^-1^	[61]
k_3b_	1.7 × 10^-3 ^sec^-1^	[61]
k_4b_, k_5b_	2.9 × 10^-3 ^sec^-1^	[34]
k_6b_	5.8 × 10^-3 ^sec^-1^	[34]

**Transport parameters**		

D_monomer_	2 × 10^-14^–2 × 10^-15 ^m^2^sec^-1^	[49, 50]

### Effect of ligand concentration on signaling reaction mechanism in A-431 cells (high receptor density)

**Figure 6:**
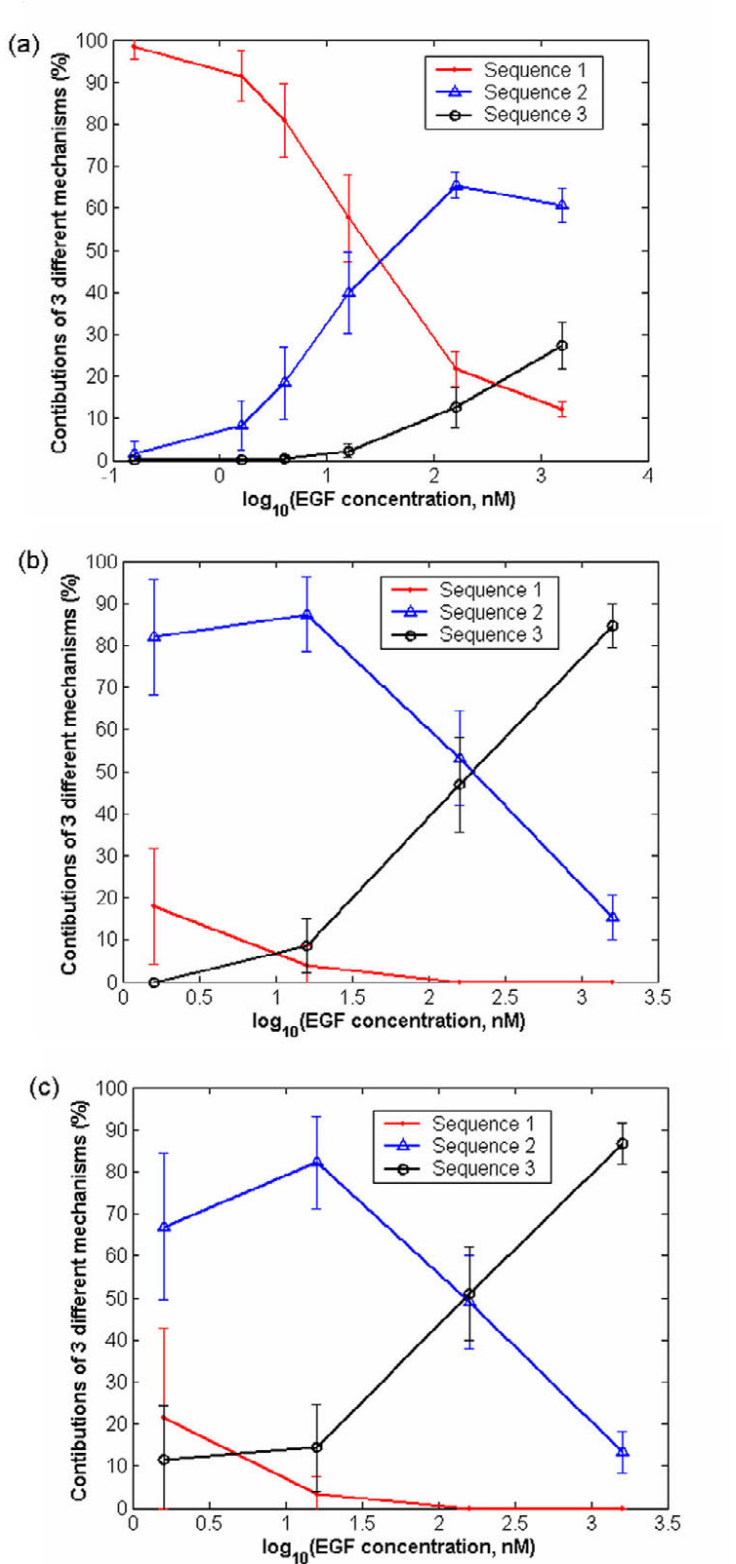
Contributions of the different reaction mechanisms at 60 sec for different concentrations of EGF with (a) a receptor number density of 5500 receptors per μm^2 ^and D = 2 × 10^-14 ^m^2^sec^-1^, (b) a receptor number density of 125 receptors per μm^2 ^and D = 2 × 10^-14 ^m^2^sec^-1^, and (c) a receptor number density of 125 receptors per μm^2 ^and D = 2 × 10^-15 ^m^2^sec^-1^.

Single particle tracking experiments [5] are typically limited to low ligand concentrations. High concentration of ligand would lead to fluorescence of a large number of EGFRs making it impossible to visualize individual particles. However, simulations can be used to elucidate the influence of extracellular EGF concentration on EGFR dimerization. Our simulations indicated that the relative contributions of sequences 1–3 at 60 sec change with ligand concentration (Fig. 6(a)). At low ligand concentration, sequence 1 dominates, whereas at higher ligand concentration, a significant fraction of dimers form via sequence 2. Furthermore, sequence 3 also occurs to appreciable extent at high concentration of EGF. At low ligand concentration, most of the ligand gets bound to dimerized receptors, which have a higher ligand affinity; however, the extent to which free EGFR dimerization can occur is limited. At higher ligand concentration, when a significant fraction of ligand is attached to monomers, the coupling between ligand attached monomer and free or ligand attached monomer gives rise to dimers. The relative contribution of the sequences also changes with time. Specifically, initial ligand binding occurs on predimerized receptors, and hence, the relative contribution of sequence 1 is higher at short times. At longer times, after binding of ligand on monomers, sequences 2 and 3 start contributing. With an increase in ligand concentration, the contributions of sequences 2 and 3 increase at a faster rate. The contribution of sequence 3 is higher at longer times after accumulation of ligand bound monomers. As a final note, the time needed to reach equilibrium substantially decreases as the concentration of ligand increases (not shown), e.g., to a total of a few sec at 160 nM. As a result, high ligand concentrations may challenge single particle tracking experiments also in terms of temporal resolution.

Support for the suggested mechanisms also comes from biochemical studies. The experimental study of [34] reported that at low doses of EGF, inhibition of high affinity binding by mAb108 can kill almost 50–100% of EGF binding, indicating that most of the early binding takes place by sequence 1 at low EGF concentration. However, this inhibition is overcome at higher concentration (~20–50 times) of EGF, which is indicative of substantial formation of EGF bound dimerized EGFR via sequence 2, consistent with the results of our simulations. A larger scale simulation with variable receptor densities in different regions of the plasma membrane will be developed in the future for quantitative comparison with such biochemical experiments. A recent equilibrium based study [13] has shown that such spatial heterogeneities have strong influence on the amount of EGF binding on EGFR, motivating a more detailed analysis of EGFR on the plasma membrane.

### Effect of ligand concentration and receptor mobility on signaling reaction mechanism in cells with normal receptor density

Two important factors influencing ligand binding and dimerization are the receptor density and receptor mobility. The receptor density can significantly influence the mechanism of EGF binding as shown in Fig. 6(b). At lower receptor density (125 receptors per μm^2^) sequence 1 occurs to a much lower extent as compared to the A-431 cells. For this lower receptor density, at lower EGF concentration sequence 2 is dominant, whereas at higher EGF concentrations, sequence 3 is dominant. Sequence 1 is not important at low receptor density, because of the low amount of EGF free dimers (negligible at the low receptor density considered in this work).

A tenfold decrease in receptor mobility (from 2 × 10^-14 ^m^2^/s to 2 × 10^-15 ^m^2^/s) leads to a very small increase in the extent of sequence 3, at the expense of sequences 1 and 2 (compare Figs. 6(b) and 6(c)). This small increase is observed only at low EGF concentration. At higher EGF concentration this increase is even smaller. Sequence 3 occurs to a larger extent at slower diffusion because dimerization is slowing down and so more monomers associate with ligand. At higher EGF concentration, this effect is not as prominent because EGF binding is faster leading to more EGF bound EGFRs, thereby increased dimerization occurs among EGF bound monomer EGFRs even with a higher receptor diffusivity.

Several studies have indicated inhomogeneities in the plasma membrane and excellent reviews have been published on this topic including [18,35-38]. These studies have suggested localization of receptors within small regions, called microdomains, in the plasma membrane. An implication of the containment of receptors in the microdomains is the observation of lower macroscopic diffusivity as has been discussed in [39]. As a result, the microscopic diffusivity can potentially be at least 1–2 orders of magnitude faster than the diffusivity reported in literature. Therefore, we have also studied the effect of a higher diffusivity. In contrast to decreasing diffusivity from 2 × 10^-15 ^m^2^/s to 2 × 10^-14 ^m^2^/s mentioned above, larger changes are observed at high ligand concentration (e.g., 1600 nM) and a receptor density of 125 receptors per μm^2 ^for a change in diffusivity from 2 × 10^-14 ^m^2^/s to 2 × 10^-13 ^m^2^/s. Specifically, the contribution of sequence 2 increases from ~15% to ~30% at the expense of sequence 3 which decreases from ~85% to ~70%. An increase in receptor diffusivity leads to an increased rate of dimerization between an occupied and a free receptor in comparison to ligand binding on a free receptor. Overall, a faster diffusivity can lead to an overall increase in the dimerization rate but this effect is not dramatic under our simulation conditions.

## Discussion

Our simulation results suggest future single particle tracking experiments or related microscopy experiments. It may be difficult to perform the single particle tracking experiments of [5] at higher ligand concentration in A-431 cells due to the difficulty in visualization of single EGFR and possibly to the short time scales over which transients are over. However, such experiments can potentially be performed in cells with a lower average receptor density. On such cells, the increased contributions of sequences 2 and 3 should be observed to further validate our model. Possible discrepancies between experiments and model could provide new insights to enhance our current understanding of the underlying signaling processes.

The variation in receptor density and receptor mobility can stem from different cell types as well as different spatial features/locations in the plasma membrane (see Methods section for references). Future microscopy experiments should be designed to observe the reaction events and transients of low and high intensity spots, as reported by [5], in different domains of the plasma membrane in the same cell. Such data can then be used to estimate the local density of the receptors which in turn can help in understanding the receptor distribution in the plasma membrane.

This work shows the influence of receptor density and receptor mobility as a biophysical control of signaling processes over the inflexible thermodynamic and biochemical properties. A key suggestion from this work is that it is not adequate to treat the receptor-receptor interactions based only on their kinetic and thermodynamic parameters. Instead, their spatial properties, such as local receptor densities and local lateral mobility, can play significant roles in determining the intracellular signaling. Herein, we have used a simplistic representation of the plasma membrane. This model can be extended to include experimentally reported anomalous hop-like diffusion [18] and other spatial features, such as clathrin coated pits, lipid rafts, caveolae and other microdomains [35,40]. Such features not only facilitate an enhanced control at the level of plasma membrane but can also be important for the wide diversity of signaling outcomes from limited varieties of ligands and receptors.

## Conclusion

We have developed a computational framework for studying cell surface receptor dynamics that can bridge biochemical data on one hand with various microscopy experiments on the other, which currently lack simultaneous high spatial and temporal resolution. This work provides an important step forward in the era of *in vivo *imaging based modeling approaches [8,41]. For example, in this work comparison of MC simulations with single particle tracking experiments reveals how the sequence of receptor-receptor and ligand-receptor reaction events depends on the ligand concentration, receptor density and receptor mobility. Our computer simulations reveal the underlying mechanism on the plasma membrane leading to dimerized and ligand (EGF) bound receptors.

Considering the interest in targeted antibodies for cancer therapeutics [42], a detailed understanding of the biochemical mechanisms involved in signal sensing at the plasma membrane is desired. Future advancements in medicine will ultimately also include the mathematical analysis and modeling of ErbB receptor diffusion, dimerization and clustering, which will increase our understanding of tumorigenesis and lead to medical advances, such as individualized therapy for heterogeneous cancers.

## Methods

### A hybrid null-event algorithm

A coarse, molecular level based computational framework that leaves atomistic details out, e.g., conformations and vibrations of proteins into potential energy surface minima, but still provides the sequence of molecular events at the receptor length scale was employed. Herein, we have utilized a kinetic, lattice MC method for simulating the EGFR dynamics. Microscopic events modeled include receptor dimerization and decomposition, ligand-receptor association and dissociation, and Brownian diffusion of receptors (see Figs. 1 and 2). Formation of high-mers that happens at longer times is not considered in this work.

The existence of multiple timescales in the system and low surface density of receptors make lattice MC simulations computationally prohibitive. We have devised a hybrid between the continuous time MC method [43] and the null-event MC method (see [44] for an overview of spatial MC algorithms) to increase the speed of the null-event algorithm but maintain its flexibility. In our hybrid null-event algorithm, only lattice sites filled with receptors were randomly selected, resulting in two to four orders of magnitude speedup, depending on receptor density, relative to a traditional null-event MC algorithm where all sites are randomly chosen. Additionally, operations that are often involved in a continuous time MC method, such as summation of transition probabilities and searches over the entire lattice, are avoided, resulting in further acceleration of simulations.

The spatial domain was represented using a two-dimensional square lattice that was initially randomly populated with a given density of receptors. Periodic boundary conditions were employed [45]. After initialization, an occupied site was randomly picked and one of the microscopic events is possibly selected to occur based on probabilities described below.

### Transition probability of diffusion

The probability of diffusion per unit time in all four directions on a square lattice was calculated using random walk theory from the diffusivity

Γd=4Da2,     (1)
 MathType@MTEF@5@5@+=feaafiart1ev1aaatCvAUfKttLearuWrP9MDH5MBPbIqV92AaeXatLxBI9gBaebbnrfifHhDYfgasaacH8akY=wiFfYdH8Gipec8Eeeu0xXdbba9frFj0=OqFfea0dXdd9vqai=hGuQ8kuc9pgc9s8qqaq=dirpe0xb9q8qiLsFr0=vr0=vr0dc8meaabaqaciGacaGaaeqabaqabeGadaaakeaacqqHtoWrdaqhaaqaaaqaaiabdsgaKbaacqGH9aqpdaWcaaqaaiabisda0iabdseaebqaaiabdggaHnaaDaaabaaabaGaeGOmaidaaaaacqGGSaalcaWLjaGaaCzcamaabmaabaGaeGymaedacaGLOaGaayzkaaaaaa@39A2@

where *a *is the microscopic lattice pixel dimension (taken here to be 2 nm) and D is the corresponding diffusivity of a receptor or a dimer. The transition probability of diffusion per unit time in moving from site *i *to site *j is*

Γi→jd=14Γdσi(1−σj)     j∈Bi     (2)
 MathType@MTEF@5@5@+=feaafiart1ev1aaatCvAUfKttLearuWrP9MDH5MBPbIqV92AaeXatLxBI9gBaebbnrfifHhDYfgasaacH8akY=wiFfYdH8Gipec8Eeeu0xXdbba9frFj0=OqFfea0dXdd9vqai=hGuQ8kuc9pgc9s8qqaq=dirpe0xb9q8qiLsFr0=vr0=vr0dc8meaabaqaciGacaGaaeqabaqabeGadaaakeaacqqHtoWrdaqhaaqaaiabdMgaPjabgkziUkabdQgaQbqaaiabdsgaKbaacqGH9aqpdaWcaaqaaiabigdaXaqaaiabisda0aaacqqHtoWrdaqhaaqaaaqaaiabdsgaKbaacqaHdpWCdaWgaaqaaiabdMgaPbqabaGaeiikaGIaeGymaeJaeyOeI0Iaeq4Wdm3aaSbaaeaacqWGQbGAaeqaaiabcMcaPiaaykW7caaMc8UaaGPaVlaaykW7caaMc8UaemOAaOMaeyicI4SaemOqai0aaSbaaSqaaiabdMgaPbqabaGccaWLjaGaaCzcamaabmaabaGaeGOmaidacaGLOaGaayzkaaaaaa@5509@

where *B*_*i *_denotes the set of sites to which diffusion from site *i *can occur. In our model, this set includes all 4 first-nearest neighboring sites. σ_*i *_is the occupancy (discrete) function that is 1, if site *i *is filled, or 0, if site *i *is empty (a single index indicating the site is herein used to simplify notation). According to Eq. (2), the transition probability of diffusion per unit time along any direction on the square lattice can be 0 or 14Γd
 MathType@MTEF@5@5@+=feaafiart1ev1aaatCvAUfKttLearuWrP9MDH5MBPbIqV92AaeXatLxBI9gBaebbnrfifHhDYfgasaacH8akY=wiFfYdH8Gipec8Eeeu0xXdbba9frFj0=OqFfea0dXdd9vqai=hGuQ8kuc9pgc9s8qqaq=dirpe0xb9q8qiLsFr0=vr0=vr0dc8meaabaqaciGacaGaaeqabaqabeGadaaakeaadaWcaaqaaiabigdaXaqaaiabisda0aaacqqHtoWrdaqhaaqaaaqaaiabdsgaKbaaaaa@317F@, depending on the occupancy of the first-nearest neighboring site in the corresponding direction.

### Transition probability of reactions

The transition probability of a reaction was obtained in terms of the macroscopic reaction rate constants, *k*. For a first-order (e.g., the decomposition of an EGFR dimer) or pseudo-first order (e.g., the EGF binding onto a receptor because EGF is assumed at a constant concentration) reaction (see Fig. 2), one has

A→C,   Γir=kσi     (3)
 MathType@MTEF@5@5@+=feaafiart1ev1aaatCvAUfKttLearuWrP9MDH5MBPbIqV92AaeXatLxBI9gBaebbnrfifHhDYfgasaacH8akY=wiFfYdH8Gipec8Eeeu0xXdbba9frFj0=OqFfea0dXdd9vqai=hGuQ8kuc9pgc9s8qqaq=dirpe0xb9q8qiLsFr0=vr0=vr0dc8meaabaqaciGacaGaaeqabaqabeGadaaakeaacqqGbbqqcqGHsgIRcqqGdbWqcqqGSaalcaaMc8UaaGPaVlaaykW7cqqHtoWrdaqhaaqaaiabdMgaPbqaaiabdkhaYbaacqGH9aqpcqWGRbWAiiaacqWFdpWCdaWgaaqaaiabdMgaPbqabaGaaCzcaiaaxMaadaqadaqaaiabiodaZaGaayjkaiaawMcaaaaa@43ED@

where Γir
 MathType@MTEF@5@5@+=feaafiart1ev1aaatCvAUfKttLearuWrP9MDH5MBPbIqV92AaeXatLxBI9gBaebbnrfifHhDYfgasaacH8akY=wiFfYdH8Gipec8Eeeu0xXdbba9frFj0=OqFfea0dXdd9vqai=hGuQ8kuc9pgc9s8qqaq=dirpe0xb9q8qiLsFr0=vr0=vr0dc8meaabaqaciGacaGaaeqabaqabeGadaaakeaacqqHtoWrdaqhaaqaaiabdMgaPbqaaiabdkhaYbaaaaa@3100@ is the transition probability of reaction at site *i*. For a bimolecular reaction on a square lattice (e.g., receptor-receptor dimerization), the transition probability per unit time at selected site *i *was modeled as:

for the reaction: A + B→C, Γir=k4σiσj,     (4)
 MathType@MTEF@5@5@+=feaafiart1ev1aaatCvAUfKttLearuWrP9MDH5MBPbIqV92AaeXatLxBI9gBaebbnrfifHhDYfgasaacH8akY=wiFfYdH8Gipec8Eeeu0xXdbba9frFj0=OqFfea0dXdd9vqai=hGuQ8kuc9pgc9s8qqaq=dirpe0xb9q8qiLsFr0=vr0=vr0dc8meaabaqaciGacaGaaeqabaqabeGadaaakeaacqqGMbGzcqqGVbWBcqqGYbGCcqqGGaaicqqG0baDcqqGObaAcqqGLbqzcqqGGaaicqqGYbGCcqqGLbqzcqqGHbqycqqGJbWycqqG0baDcqqGPbqAcqqGVbWBcqqGUbGBcqqG6aGocqqGGaaicqqGbbqqcqqGGaaicqqGRaWkcqqGGaaicqqGcbGqcqGHsgIRieaacqWFdbWqcqGGSaalcqqGGaaicqqHtoWrdaqhaaqaaiab=LgaPbqaaiab=jhaYbaacqGH9aqpdaWcaaqaaiab=TgaRbqaaiabisda0aaaiiaacqGFdpWCdaWgaaqaaiab=LgaPbqabaGae43Wdm3aaSbaaeaacqWFQbGAaeqaaiabcYcaSiaaxMaacaWLjaWaaeWaaeaacqaI0aanaiaawIcacaGLPaaaaaa@5F0B@

and for the reaction: 2A→C, Γir=k2σiσj.     (5)
 MathType@MTEF@5@5@+=feaafiart1ev1aaatCvAUfKttLearuWrP9MDH5MBPbIqV92AaeXatLxBI9gBaebbnrfifHhDYfgasaacH8akY=wiFfYdH8Gipec8Eeeu0xXdbba9frFj0=OqFfea0dXdd9vqai=hGuQ8kuc9pgc9s8qqaq=dirpe0xb9q8qiLsFr0=vr0=vr0dc8meaabaqaciGacaGaaeqabaqabeGadaaakeaacqqGHbqycqqGUbGBcqqGKbazcqqGGaaicqqGMbGzcqqGVbWBcqqGYbGCcqqGGaaicqqG0baDcqqGObaAcqqGLbqzcqqGGaaicqqGYbGCcqqGLbqzcqqGHbqycqqGJbWycqqG0baDcqqGPbqAcqqGVbWBcqqGUbGBcqqG6aGocqqGGaaicqqGYaGmcqqGbbqqcqGHsgIRcqqGdbWqcqqGSaalcqqGGaaicqqHtoWrdaqhaaqaaGqaaiab=LgaPbqaaiab=jhaYbaacqGH9aqpdaWcaaqaaiab=TgaRbqaaiabikdaYaaaiiaacqGFdpWCdaWgaaqaaiab=LgaPbqabaGae43Wdm3aaSbaaeaacqWFQbGAaeqaaiabc6caUiaaxMaacaWLjaWaaeWaaeaacqaI1aqnaiaawIcacaGLPaaaaaa@6145@

Here the reacting species (A and B or A and A) occupy adjacent sites *i *and *j*, and the units of *k *are (molecules/site)^-1^sec^-1^. The factor of four in *k*/4 in Eq. (4) accounts for the fact that all four neighboring sites of site i were randomly chosen to search for the existence of species B. Similarly, the factor of 2 in Eq. 5 was due to the degeneracy of species participating in the homodimerization.

### Event selection and time advancement

After an occupied site, say *i*, was selected, the transition probabilities per unit time of all possible events were computed. The probability for a certain event '*x*' at site *i *was calculated as

pix=ΓixΓmax⁡,     (6)
 MathType@MTEF@5@5@+=feaafiart1ev1aaatCvAUfKttLearuWrP9MDH5MBPbIqV92AaeXatLxBI9gBaebbnrfifHhDYfgasaacH8akY=wiFfYdH8Gipec8Eeeu0xXdbba9frFj0=OqFfea0dXdd9vqai=hGuQ8kuc9pgc9s8qqaq=dirpe0xb9q8qiLsFr0=vr0=vr0dc8meaabaqaciGacaGaaeqabaqabeGadaaakeaacqWGWbaCdaqhaaqaaiabdMgaPbqaaiabdIha4baacqGH9aqpdaWcaaqaaiabfo5ahnaaDaaabaGaemyAaKgabaGaemiEaGhaaaqaaiabfo5ahnaaBaaabaGagiyBa0MaeiyyaeMaeiiEaGhabeaaaaGaeiilaWIaaCzcaiaaxMaadaqadaqaaiabiAda2aGaayjkaiaawMcaaaaa@40D7@

where Γ_max _is a normalization constant to ensure that the selection probability of each event is always less than or equal to 1 and Γix
 MathType@MTEF@5@5@+=feaafiart1ev1aaatCvAUfKttLearuWrP9MDH5MBPbIqV92AaeXatLxBI9gBaebbnrfifHhDYfgasaacH8akY=wiFfYdH8Gipec8Eeeu0xXdbba9frFj0=OqFfea0dXdd9vqai=hGuQ8kuc9pgc9s8qqaq=dirpe0xb9q8qiLsFr0=vr0=vr0dc8meaabaqaciGacaGaaeqabaqabeGadaaakeaacqqHtoWrdaqhaaqaaiabdMgaPbqaaiabdIha4baaaaa@310C@ is the transition probability of event *x *(reaction or diffusion) at site *i*, as defined above. The maximum values of transition probabilities per unit time of events described by Eqs. (2)-(4) are Γ^d^/4, k, k/4, and k/2, respectively. We defined the normalization constant as

Γmax⁡=4(Γd4+max⁡{∑all forward reaction eventsΓr}),+max⁡{∑all backward reaction eventsΓr}
 MathType@MTEF@5@5@+=feaafiart1ev1aaatCvAUfKttLearuWrP9MDH5MBPbIqV92AaeXatLxBI9gBaebbnrfifHhDYfgasaacH8akY=wiFfYdH8Gipec8Eeeu0xXdbba9frFj0=OqFfea0dXdd9vqai=hGuQ8kuc9pgc9s8qqaq=dirpe0xb9q8qiLsFr0=vr0=vr0dc8meaabaqaciGacaGaaeqabaqabeGadaaakqaabeqaaiabfo5ahnaaBaaabaGagiyBa0MaeiyyaeMaeiiEaGhabeaacqGH9aqpcqaI0aandaqadaqaamaalaaabaGaeu4KdC0aaWbaaeqabaGaemizaqgaaaqaaiabisda0aaacqGHRaWkcyGGTbqBcqGGHbqycqGG4baEdaGadaqaamaaqafabaGaeu4KdC0aaWbaaeqabaGaemOCaihaaaqaaiabdggaHjabdYgaSjabdYgaSjabbccaGiabdAgaMjabd+gaVjabdkhaYjabdEha3jabdggaHjabdkhaYjabdsgaKjabbccaGiabdkhaYjabdwgaLjabdggaHjabdogaJjabdsha0jabdMgaPjabd+gaVjabd6gaUjabbccaGiabdwgaLjabdAha2jabdwgaLjabd6gaUjabdsha0jabdohaZbqabiabggHiLdaacaGL7bGaayzFaaaacaGLOaGaayzkaaGamaiGaaaqciilaWcabaGaey4kaSIagiyBa0MaeiyyaeMaeiiEaG3aaiWaaeaadaaeqbqaaiabfo5ahnaaCaaabeqaaiabdkhaYbaaaeaacqWGHbqycqWGSbaBcqWGSbaBcqqGGaaicqWGIbGycqWGHbqycqWGJbWycqWGRbWAcqWG3bWDcqWGHbqycqWGYbGCcqWGKbazcqqGGaaicqWGYbGCcqWGLbqzcqWGHbqycqWGJbWycqWG0baDcqWGPbqAcqWGVbWBcqWGUbGBcqqGGaaicqWGLbqzcqWG2bGDcqWGLbqzcqWGUbGBcqWG0baDcqWGZbWCaeqacqGHris5aaGaay5Eaiaaw2haaaaaaa@9C41@

where multiplication by a factor of 4 accounts for the microscopic process in each of the four directions on the square lattice. For the reaction events given in Fig. 2

Γmax⁡=Γd+4(k1f2+k2f4+k3f2)+∑i=46kif+∑i=16kib,     (7)
 MathType@MTEF@5@5@+=feaafiart1ev1aaatCvAUfKttLearuWrP9MDH5MBPbIqV92AaeXatLxBI9gBaebbnrfifHhDYfgasaacH8akY=wiFfYdH8Gipec8Eeeu0xXdbba9frFj0=OqFfea0dXdd9vqai=hGuQ8kuc9pgc9s8qqaq=dirpe0xb9q8qiLsFr0=vr0=vr0dc8meaabaqaciGacaGaaeqabaqabeGadaaakeaacqqHtoWrdaWgaaqaaiGbc2gaTjabcggaHjabcIha4bqabaGaeyypa0Jaeu4KdC0aaWbaaeqabaGaemizaqgaaiabgUcaRiabisda0maabmaabaWaaSaaaeaacqWGRbWAdaWgaaqaaiabigdaXiabdAgaMbqabaaabaGaeGOmaidaaiabgUcaRmaalaaabaGaem4AaS2aaSbaaeaacqaIYaGmcqWGMbGzaeqaaaqaaiabisda0aaacqGHRaWkdaWcaaqaaiabdUgaRnaaBaaabaGaeG4mamJaemOzaygabeaaaeaacqaIYaGmaaaacaGLOaGaayzkaaGaey4kaSYaaabCaeaacqWGRbWAdaWgaaqaaiabdMgaPjabdAgaMbqabaaabaGaemyAaKMaeyypa0JaeGinaqdabaGaeGOnaydacqGHris5aiabgUcaRmaaqahabaGaem4AaS2aaSbaaeaacqWGPbqAcqWGIbGyaeqaaaqaaiabdMgaPjabg2da9iabigdaXaqaaiabiAda2aGaeyyeIuoacqGGSaalcaWLjaGaaCzcamaabmaabaGaeG4naCdacaGLOaGaayzkaaaaaa@6577@

where the subscripts *f *and *b *refer to the forward and backward reaction events, respectively, and *i *denotes the corresponding reaction according to Fig. 2. A random number '*r*' was finally chosen from a uniform distribution between 0 and 1. The events were randomly ranked. The smallest value of *m *satisfying ∑x=1mpix>r
 MathType@MTEF@5@5@+=feaafiart1ev1aaatCvAUfKttLearuWrP9MDH5MBPbIqV92AaeXatLxBI9gBaebbnrfifHhDYfgasaacH8akY=wiFfYdH8Gipec8Eeeu0xXdbba9frFj0=OqFfea0dXdd9vqai=hGuQ8kuc9pgc9s8qqaq=dirpe0xb9q8qiLsFr0=vr0=vr0dc8meaabaqaciGacaGaaeqabaqabeGadaaakeaadaaeWbqaaiabdchaWnaaDaaabaGaemyAaKgabaGaemiEaGhaaaqaaiabdIha4jabg2da9iabigdaXaqaaiabd2gaTbGaeyyeIuoacqGH+aGpcqWGYbGCaaa@3A7E@ criterion was chosen. If no value of *m *satisfied the above criterion, then no event was selected to occur (a null-event) and a new occupied site was again randomly picked. Otherwise, the *m*^*th *^event was selected, the populations on the lattice were updated accordingly to reflect the stoichiometry of the reaction or diffusion process, and the real time was advanced based on the most frequently selected event as suggested in [44]. In our simulations, real time was advanced based on the diffusion of receptors according to which the average time step after each successful diffusion event was calculated as

Δt=1∑i=1No.ofsites(σi∑j∈BiΓi→jd(1-σj))=114Γd∑occupiedsites(∑j∈Bi(1-σj)).     (8)
 MathType@MTEF@5@5@+=feaafiart1ev1aaatCvAUfKttLearuWrP9MDH5MBPbIqV92AaeXatLxBI9gBaebbnrfifHhDYfgasaacH8akY=wiFfYdH8Gipec8Eeeu0xXdbba9frFj0=OqFfea0dXdd9vqai=hGuQ8kuc9pgc9s8qqaq=dirpe0xb9q8qiLsFr0=vr0=vr0dc8meaabaqaciGacaGaaeqabaqabeGadaaakeaaiiaacqWFuoarieaacqGF0baDcqGF9aqpdaWcaaqaaiab+fdaXaqaamaaqahabaWaaeWaaeaacqWFdpWCdaWgaaqaaiab+LgaPbqabaWaaabCaeaacqWFtoWrdaqhaaqaaiab+LgaPjabgkziUkab+PgaQbqaaiab+rgaKbaadaqadaqaaiab+fdaXiab+1caTiab=n8aZnaaBaaaleaacqGFQbGAaeqaaaGccaGLOaGaayzkaaaabaGae4NAaOMae8hcI4Sae4Nqai0aaSbaaSqaaiab+LgaPbqabaaakeaaaiabggHiLdaacaGLOaGaayzkaaaabaGae4xAaKMae4xpa0Jae4xmaedabaGae4Nta4Kae43Ba8Mae4Nla4Iae4hiaaIae43Ba8Mae4NzayMae4hiaaIae43CamNae4xAaKMae4hDaqNae4xzauMae43CamhacqGHris5aaaacqGF9aqpdaWcaaqaaiab+fdaXaqaamaalaaabaGae4xmaedabaGae4hnaqdaaiab=n5ahnaaCaaabeqaaiab+rgaKbaadaaeWbqaamaabmaabaWaaabCaeaadaqadaqaaiab+fdaXiab+1caTiab=n8aZnaaBaaaleaacqGFQbGAaeqaaaGccaGLOaGaayzkaaaabaGae4NAaOMae8hcI4Sae4Nqai0aaSbaaSqaaiab+LgaPbqabaaakeaaaiabggHiLdaacaGLOaGaayzkaaaabaGae43Ba8Mae43yamMae43yamMae4xDauNae4hCaaNae4xAaKMae4xzauMae4hzaqMae4hiaaIae43CamNae4xAaKMae4hDaqNae4xzauMae43CamhabaaacqGHris5aaaacqGFGaaicqGFUaGlcaWLjaGaaCzcamaabmaabaGae4hoaGdacaGLOaGaayzkaaaaaa@9004@

The summation in the denominator was updated each time a successful event happens by subtracting the previous occupancy values affected by the selected event and adding the new ones. In this way, the summation was carried out only once (at the beginning of a simulation) over all occupied sites. Finally, a new site is picked randomly until the desired real time is reached.

The hybrid null-event MC algorithm explained above was implemented (for details on the null-event algorithm see [44]) using Fortran 90. For each data point, 10 simulations with different seeds of the random number generator were used to collect statistics.

### Simulation size and model parameters

In this work, the cell surface was represented using a 2-dimensional square lattice with each pixel being 2 nm × 2 nm in size. The number density of receptors ranges from ~10^2 ^receptors per μm^2 ^on normal cells [46] to ~10^3 ^receptors per μm^2 ^on human epithelioid carcinoma cells (A-431 cells) which overexpress EGFR [2]. However, the local density of receptors can be much higher *in-vivo *because of the localization of receptors in certain regions of the plasma membrane, such as in lipid rafts [35,47,48]. We simulated a low density (to represent normal cells) of 31 receptors on 500 nm × 500 nm mesh and a high density (to represent A-431 cells) of 55 receptors on 100 nm × 100 nm mesh, which are equivalent to receptor number densities of 125 and 5500 per μm^2^, respectively. The diffusivity of monomer EGFR has been reported to be around 2 × 10^-14 ^m^2^s^-1 ^[49,50]. Lower macroscopic diffusivities for EGFR have also been observed [49], which may be due to containment within cytoskeletal elements [51] or lipid rafts [52]. Based on these suggestions, the effect of a slower diffusivity is also analyzed by considering a diffusivity of 2 × 10^-15 ^m^2^s^-1^. Simulations have been performed in the 0–60 sec time interval to capture the initial transients.

The model parameters are summarized in Table 1. We assumed two types of EGF binding on the cell surface: low affinity binding (on monomer EGFR) and high affinity binding (on dimerized EGFR). Experimental information suggests the existence of a high affinity (for receptor-ligand association) receptor population, most of which, if not all, is present in the form of dimers; see review by [1]. A recent equilibrium study has also shown that this interpretation is consistent with the experimentally reported concave-up shape of the Scatchard plot [13].

Experimental studies have provided evidence of predimerized receptors on A-431 cells to different extents [9,53-56]. Consequently, a fraction (~82%) of receptors was initially placed at random locations as monomers and the remaining as dimers on simulated A-431 cells for comparison with single particle tracking data. Corresponding to the dimerization equilibrium constant for this data, we found that at the lower receptor number density of 125 per μm^2^, there is negligible number of dimers in the absence of ligand. The receptor dimerization constants vary with ligand occupancy. Several experimental studies have shown that dimerization between unbounded receptors occurs with lower affinity than that between one bounded and one unbounded receptor. Finally, dimerization between two ligand bounded receptors occurs with the highest affinity [6,57].

## Appendix

**Figure 7:**
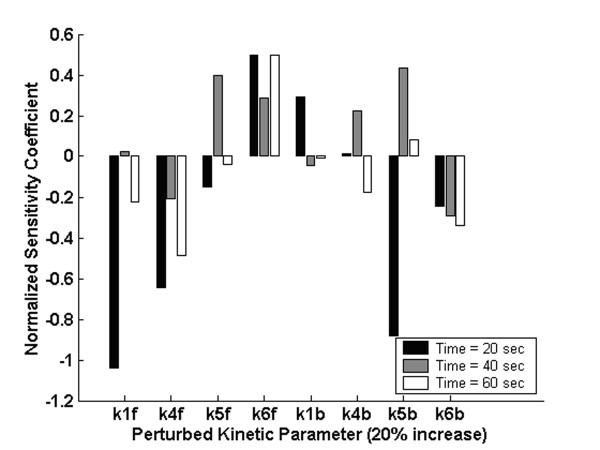
Normalized sensitivity coefficients at 3 different times (20, 40 and 60 sec) calculated by introducing a 20% increase in the kinetic parameter indicated on the x-axis.

A sensitivity analysis was performed in which each kinetic parameter (*k*_*i*_, *i = 1f, 4f, 5f, 6f, 1b, 4b, 5b, 6b*) was increased by 20%, and the change in the high intensity spots was observed at three different times (20, 40 and 60 sec) from the mean of 10 independent MC simulations. The normalized sensitivity coefficient, reported in Fig. 7, is defined as

(I−Io)/Io(ki−kio)/kio=I−Io0.2Io,     (A1)
 MathType@MTEF@5@5@+=feaafiart1ev1aqatCvAUfKttLearuWrP9MDH5MBPbIqV92AaeXatLxBI9gBaebbnrfifHhDYfgasaacH8akY=wiFfYdH8Gipec8Eeeu0xXdbba9frFj0=OqFfea0dXdd9vqai=hGuQ8kuc9pgc9s8qqaq=dirpe0xb9q8qiLsFr0=vr0=vr0dc8meaabaqaciGacaGaaeqabaqabeGadaaakeaadaWcaaqaamaalyaabaGaeiikaGIaemysaKKaeyOeI0IaemysaK0aaSbaaSqaaiabd+gaVbqabaGccqGGPaqkaeaacqWGjbqsdaWgaaWcbaGaem4Ba8gabeaaaaaakeaadaWcgaqaaiabcIcaOiabdUgaRnaaBaaaleaacqWGPbqAaeqaaOGaeyOeI0Iaem4AaS2aaSbaaSqaaiabdMgaPjabd+gaVbqabaGccqGGPaqkaeaacqWGRbWAdaWgaaWcbaGaemyAaKMaem4Ba8gabeaaaaaaaOGaeyypa0ZaaSaaaeaacqWGjbqscqGHsislcqWGjbqsdaWgaaWcbaGaem4Ba8gabeaaaOqaaiabicdaWiabc6caUiabikdaYiabdMeajnaaBaaaleaacqWGVbWBaeqaaaaakiabcYcaSiaaxMaacaWLjaWaaeWaaeaacqqGbbqqcqqGXaqmaiaawIcacaGLPaaaaaa@5543@

where I is the % of high intensity spots (y axis in Fig. 4a) upon perturbing the kinetic parameter, k_i_, and I_o _is the nominal value corresponding to the original set of kinetic parameters, k_io_. Only 8 of the 12 kinetic parameters were independently perturbed because of the two equilibrium constrains reported in [14], i.e.,

K2=K1K5K4, and      (A2)
 MathType@MTEF@5@5@+=feaafiart1ev1aqatCvAUfKttLearuWrP9MDH5MBPbIqV92AaeXatLxBI9gBaebbnrfifHhDYfgasaacH8akY=wiFfYdH8Gipec8Eeeu0xXdbba9frFj0=OqFfea0dXdd9vqai=hGuQ8kuc9pgc9s8qqaq=dirpe0xb9q8qiLsFr0=vr0=vr0dc8meaabaqaciGacaGaaeqabaqabeGadaaakeaacqWGlbWsdaWgaaWcbaGaeGOmaidabeaakiabg2da9maalaaabaGaem4saS0aaSbaaSqaaiabigdaXaqabaGccqWGlbWsdaWgaaWcbaGaeGynaudabeaaaOqaaiabdUealnaaBaaaleaacqaI0aanaeqaaaaakiabcYcaSiabbccaGiabbggaHjabb6gaUjabbsgaKjabbccaGiaaxMaacaWLjaWaaeWaaeaacqqGbbqqcqqGYaGmaiaawIcacaGLPaaaaaa@4213@

K3=K1K5K6(K4)2.     (A3)
 MathType@MTEF@5@5@+=feaafiart1ev1aqatCvAUfKttLearuWrP9MDH5MBPbIqV92AaeXatLxBI9gBaebbnrfifHhDYfgasaacH8akY=wiFfYdH8Gipec8Eeeu0xXdbba9frFj0=OqFfea0dXdd9vqai=hGuQ8kuc9pgc9s8qqaq=dirpe0xb9q8qiLsFr0=vr0=vr0dc8meaabaqaciGacaGaaeqabaqabeGadaaakeaacqWGlbWsdaWgaaWcbaGaeG4mamdabeaakiabg2da9maalaaabaGaem4saS0aaSbaaSqaaiabigdaXaqabaGccqWGlbWsdaWgaaWcbaGaeGynaudabeaakiabdUealnaaBaaaleaacqaI2aGnaeqaaaGcbaWaaeWaaeaacqWGlbWsdaWgaaWcbaGaeGinaqdabeaaaOGaayjkaiaawMcaamaaCaaaleqabaGaeGOmaidaaaaakiabc6caUiaaxMaacaWLjaWaaeWaaeaacqqGbbqqcqqGZaWmaiaawIcacaGLPaaaaaa@4193@

The equilibrium relations determine the changes in the kinetic parameters of the dependent reactions 2 and 3 (see Fig. 2) upon perturbing those of the linearly independent reactions. A change in an equilibrium constant can be associated with a change in the forward, backward, or both rate constants. For simplicity a change in the rate constant of a forward (backward) linearly independent reaction is taken to cause a change in the forward (backward) rate constant of the linearly dependent reactions. Specifically, one has

k2f=(k2f)ofk1ffk5ffk4f,     (A4)
 MathType@MTEF@5@5@+=feaafiart1ev1aqatCvAUfKttLearuWrP9MDH5MBPbIqV92AaeXatLxBI9gBaebbnrfifHhDYfgasaacH8akY=wiFfYdH8Gipec8Eeeu0xXdbba9frFj0=OqFfea0dXdd9vqai=hGuQ8kuc9pgc9s8qqaq=dirpe0xb9q8qiLsFr0=vr0=vr0dc8meaabaqaciGacaGaaeqabaqabeGadaaakeaacqWGRbWAdaWgaaWcbaGaeGOmaiJaemOzaygabeaakiabg2da9maabmaabaGaem4AaS2aaSbaaSqaaiabikdaYiabdAgaMbqabaaakiaawIcacaGLPaaadaWgaaWcbaGaem4Ba8gabeaakmaalaaabaGaemOzay2aaSbaaSqaaiabdUgaRnaaBaaameaacqaIXaqmcqWGMbGzaeqaaaWcbeaakiabdAgaMnaaBaaaleaacqWGRbWAdaWgaaadbaGaeGynauJaemOzaygabeaaaSqabaaakeaacqWGMbGzdaWgaaWcbaGaem4AaS2aaSbaaWqaaiabisda0iabdAgaMbqabaaaleqaaaaakiabcYcaSiaaxMaacaWLjaWaaeWaaeaacqqGbbqqcqqG0aanaiaawIcacaGLPaaaaaa@4E8B@

k3f=(k3f)ofk1ffk5ffk6f(fk4f)2,     (A5)
 MathType@MTEF@5@5@+=feaafiart1ev1aqatCvAUfKttLearuWrP9MDH5MBPbIqV92AaeXatLxBI9gBaebbnrfifHhDYfgasaacH8akY=wiFfYdH8Gipec8Eeeu0xXdbba9frFj0=OqFfea0dXdd9vqai=hGuQ8kuc9pgc9s8qqaq=dirpe0xb9q8qiLsFr0=vr0=vr0dc8meaabaqaciGacaGaaeqabaqabeGadaaakeaacqWGRbWAdaWgaaWcbaGaeG4mamJaemOzaygabeaakiabg2da9maabmaabaGaem4AaS2aaSbaaSqaaiabiodaZiabdAgaMbqabaaakiaawIcacaGLPaaadaWgaaWcbaGaem4Ba8gabeaakmaalaaabaGaemOzay2aaSbaaSqaaiabdUgaRnaaBaaameaacqaIXaqmcqWGMbGzaeqaaaWcbeaakiabdAgaMnaaBaaaleaacqWGRbWAdaWgaaadbaGaeGynauJaemOzaygabeaaaSqabaGccqWGMbGzdaWgaaWcbaGaem4AaS2aaSbaaWqaaiabiAda2iabdAgaMbqabaaaleqaaaGcbaWaaeWaaeaacqWGMbGzdaWgaaWcbaGaem4AaS2aaSbaaWqaaiabisda0iabdAgaMbqabaaaleqaaaGccaGLOaGaayzkaaWaaWbaaSqabeaacqaIYaGmaaaaaOGaeiilaWIaaCzcaiaaxMaadaqadaqaaiabbgeabjabbwda1aGaayjkaiaawMcaaaaa@56B4@

k2b=(k2b)ofk1bfk5bfk4b, and      (A6)
 MathType@MTEF@5@5@+=feaafiart1ev1aqatCvAUfKttLearuWrP9MDH5MBPbIqV92AaeXatLxBI9gBaebbnrfifHhDYfgasaacH8akY=wiFfYdH8Gipec8Eeeu0xXdbba9frFj0=OqFfea0dXdd9vqai=hGuQ8kuc9pgc9s8qqaq=dirpe0xb9q8qiLsFr0=vr0=vr0dc8meaabaqaciGacaGaaeqabaqabeGadaaakeaacqWGRbWAdaWgaaWcbaGaeGOmaiJaemOyaigabeaakiabg2da9maabmaabaGaem4AaS2aaSbaaSqaaiabikdaYiabdkgaIbqabaaakiaawIcacaGLPaaadaWgaaWcbaGaem4Ba8gabeaakmaalaaabaGaemOzay2aaSbaaSqaaiabdUgaRnaaBaaameaacqaIXaqmcqWGIbGyaeqaaaWcbeaakiabdAgaMnaaBaaaleaacqWGRbWAdaWgaaadbaGaeGynauJaemOyaigabeaaaSqabaaakeaacqWGMbGzdaWgaaWcbaGaem4AaS2aaSbaaWqaaiabisda0iabdkgaIbqabaaaleqaaaaakiabcYcaSiabbccaGiabbggaHjabb6gaUjabbsgaKjabbccaGiaaxMaacaWLjaWaaeWaaeaacqqGbbqqcqqG2aGnaiaawIcacaGLPaaaaaa@53F0@

k3b=(k3b)ofk1bfk5bfk6b(fk4b)2,     (A7)
 MathType@MTEF@5@5@+=feaafiart1ev1aqatCvAUfKttLearuWrP9MDH5MBPbIqV92AaeXatLxBI9gBaebbnrfifHhDYfgasaacH8akY=wiFfYdH8Gipec8Eeeu0xXdbba9frFj0=OqFfea0dXdd9vqai=hGuQ8kuc9pgc9s8qqaq=dirpe0xb9q8qiLsFr0=vr0=vr0dc8meaabaqaciGacaGaaeqabaqabeGadaaakeaacqWGRbWAdaWgaaWcbaGaeG4mamJaemOyaigabeaakiabg2da9maabmaabaGaem4AaS2aaSbaaSqaaiabiodaZiabdkgaIbqabaaakiaawIcacaGLPaaadaWgaaWcbaGaem4Ba8gabeaakmaalaaabaGaemOzay2aaSbaaSqaaiabdUgaRnaaBaaameaacqaIXaqmcqWGIbGyaeqaaaWcbeaakiabdAgaMnaaBaaaleaacqWGRbWAdaWgaaadbaGaeGynauJaemOyaigabeaaaSqabaGccqWGMbGzdaWgaaWcbaGaem4AaS2aaSbaaWqaaiabiAda2iabdkgaIbqabaaaleqaaaGcbaWaaeWaaeaacqWGMbGzdaWgaaWcbaGaem4AaS2aaSbaaWqaaiabisda0iabdkgaIbqabaaaleqaaaGccaGLOaGaayzkaaWaaWbaaSqabeaacqaIYaGmaaaaaOGaeiilaWIaaCzcaiaaxMaadaqadaqaaiabbgeabjabbEda3aGaayjkaiaawMcaaaaa@5688@

where subscript '*o*' denotes the nominal value, and fki
 MathType@MTEF@5@5@+=feaafiart1ev1aqatCvAUfKttLearuWrP9MDH5MBPbIqV92AaeXatLxBI9gBaebbnrfifHhDYfgasaacH8akY=wiFfYdH8Gipec8Eeeu0xXdbba9frFj0=OqFfea0dXdd9vqai=hGuQ8kuc9pgc9s8qqaq=dirpe0xb9q8qiLsFr0=vr0=vr0dc8meaabaqaciGacaGaaeqabaqabeGadaaakeaacqWGMbGzdaWgaaWcbaGaem4AaS2aaSbaaWqaaiabdMgaPbqabaaaleqaaaaa@3122@ is 1, if the kinetic parameter (*k*_*i*_) is not perturbed, and 1.2, if the parameter is increased by 20%.

## Authors' contributions

KM carried out the simulations and drafted the manuscript. DGV and JSE edited the manuscript. All authors participated in the analysis of the data. All authors read and approved the final manuscript.
